# Clinical and ultrasonographic evaluation of uninjured dominant shoulder in amateur rugby players vs a control group: a pilot study

**DOI:** 10.1007/s40477-024-00897-6

**Published:** 2024-04-06

**Authors:** Giovanni Monteleone, Alfonso Tramontana, Roberto Sorge

**Affiliations:** 1 Department of Biomedicine and Prevention, Faculty of Medicine and Surgery, University of Rome “Tor Vergata”, Rome, Italy Via Montpellier 1, 00133; 2 School of Sport and Exercise Sciences, University of Rome “Tor Vergata”, Rome, Italy; 3 Rey Juan Carlos University Madrid Spain Physical Medicine 81 and Rehabilitation, Calle tulipan s/n, Mostoles, 28933 Madrid, Spain; 4 Department of Systems Medicine, Faculty of Medicine and Surgery, University of Rome “Tor Vergata”, Rome, Italy

**Keywords:** Clinical exam of the shoulder, Rotator cuff injuries, Rugby, Shoulder injuries, Tendon calcification, Ultrasonography

## Abstract

**Background:**

Rugby is a sport involving a great number of shoulder collisions. Traumatic stress of the shoulder can weaken the static stabilizers and promote major injuries as dislocation or full-thickness tears of the rotator cuff. The goal of this study is to evaluate the clinical and ultrasonographic dominant shoulder factures in a group of amateur rugby players, with no history of shoulder injuries, and to compare them with those of a control group.

**Methods:**

52 male subjects join in the study: 26 amateur rugby players and 26 subjects, which did not practice rugby or competitive sport. Clinical history was obtained from all subjects, followed by dominant shoulder physical and ultrasonographic exams.

**Results:**

Rugby players showed a higher prevalence of positive clinical test, suggesting subacromial impingement than control group (*p* = 0.01).

Among rugby group, five players (19,2%) showed positive test for radiculopathy (*p* = 0,02), and ten players (73,1%) reported shoulder pain needing pain-reliever drugs at list one time in the last six months (*p* = 0.001). In rugby group, ultrasound exams showed 23,1% degenerative changes and 30,8% tendon calcifications in supraspinatus tendons (*p* < 0.05).

**Conclusions:**

Uninjured dominant shoulder of rugby players shows higher prevalence of clinical and ultrasound changes compare to control. Some rugby players without history of cervical symptoms show positive clinical test of cervical radiculopathy. Clinical and ultrasonographic monitoring of the shoulder can play a role in prevention and knowledge of silent shoulder damage in these athletes.

## Introduction

Rugby is a popular team sport at high risk of contact injuries: as reported by King et al. [1], the overall pooled injury incidence in male players is 99.0 per 1000 match-hr and s 11.7 per 1000 training-hr.

In rugby, shoulder injuries are mostly of traumatic origin, such as glenoid labrum tear (ie. superior labral tear) injuries caused by anterior dislocation events (i.e. Bankart lesion), rotator cuff tears and acromioclavicular joint injuries [2, 3]. In male amateur players, shoulder injuries have an incidence rate of 7.5 per 1000 player-hours [4].

Shoulder injuries as subacromial impingement, rotator cuff tendinosis, tendon inflammation, which are common in overhead sports, have reported with low frequencies in rugby [5].

These injuries arise subtly, can persist and cause abnormal shoulder kinematics, shoulder dysfunction, or shoulder pain and soreness [6].

Further injuries may affect the shoulder function: i.e. transient lesions of the cervical nerve roots or brachial plexus can cause scapular dyskinesia following by slow onset of shoulder dysfunction [7].

Furthermore, the definition of injury most frequently adopted in epidemiological studies on rugby [8] is: “Any physical complaint, which was caused by a transfer of energy that exceeded the body’s ability to maintain its structural and/or functional integrity, that was sustained by a player during a rugby match or rugby training, irrespective of the need for medical attention or time-loss from rugby activities, which occur during a match or training”.

This definition does not allow distinguishing those injuries gradually displaying, because of a collection of exposures to repeated quantity of kinetic energy [9].

Both shoulders can undergo impacts not only by accident: in both active and passive shoulder tackle, the shoulder is the first part of the player's body contacting the opponent. In the tackle, the glenohumeral joint repeatedly absorbs a large amount of kinetic energy [10].

As reported by Tambe et al. [11], the sum of traumatic stresses such as those resulting from tackles scrumms or mauls, has been indicated as a factor capable of weakening the shoulder static stabilizers.

The excessive translation of the joint parts causes an increasing stress to the rotator cuff. This extra stress is due to the increasing effort of the rotator cuff itself in supporting the failure of the static capsuloligamentous structures [12]. A great number of collisions may promote full-thickness tears of the rotator cuff or major injuries.

Some tendon and ligament changes can therefore occurring and come before other worse pathological conditions.

The ultrasound examination allows viewing changes of the tendon, and dynamically examining glenohumeral joint.

The purpose of this research is to search for clinical and ultrasound abnormalities of the uninjured dominant shoulder in healthy amateur rugby players, and to compare the findings with those of a control group not practicing rugby or competitive sport.

We hypothesized that amateur rugby players with no diagnosis of shoulder injury show different prevalence of shoulder clinical and ultrasound abnormalities than control group.

## Materials and methods

### Study population

The study involved 52 male subjects: 26 rugby players (14 militant athletes in the C1 and 12 championship in the C2 championship); 26 subjects who had never practiced rugby and who are not practicing competitive sport. The average age was 22.3 ± 1.7 years. The subjects who have undergone fracture, dislocation, shoulder surgery, or who have had any shoulder disease diagnosis have been excluded. To avoid confounding influence of rotator cuff changes correlated to age [13] all study participants were under the age of 26.

Data were collected by a self-administered questionnaire completed by each subject to get demographic information and data about rugby practice, weekly training time etc.

The study was approved by the Institutional Review Board of the University of Rome Tor Vergata and was conducted in conformity with the ethical and humane principles of research.

### Clinical evaluation

All participants were interviewed to obtain data regarding dominant shoulder, clinical history and history of shoulder pathology, including use of medication, previous direct shoulder trauma and presence of pain at rest and with use. The subjects' weights and heights were recorded. Then an orthopedic surgeon (GM) performed a physical examination:

The presence of shoulder swelling, muscle atrophy, asymmetry, and deformity were assessed. Deltoid muscle and acromion clavicular joint have palpated to find tenderness/pain areas. The following tests have performed:

#### Rotator cuff assessment

*Jobe test* (supraspinatus muscle): considered positive when downward pressure applied to the arms while they are kept abducted and internally rotated with thumbs down results in significant asymmetry in the force.

*Infraspinatus muscle strength test* (infraspinatus muscle): considered positive when there is a reduction of strength in external rotation while the arm is positioned along the side in contact with the chest and the elbow is flexed at 90°.

*Lift off test*: (subscapularis muscle) the integrity of the subscapularis muscle is evaluated by the ability to actively lift the hand away from the back and resist force.

*Napoleon test* (subscapularis muscle): performed by asking the patient to bring the elbows anteriorly while pressing the hands on the abdomen; allows muscle strength to be graded.

*Speed test* (biceps tendinitis): considered positive when pain is reproduced by keeping the shoulder in 90° of flexion, the elbow extended and the hand supinated with applied resistance.

#### Impingement assessment

*Hawkins test* (subacromial impingement): considered positive when pain is reproduced as the arm is passively raised in 90° of forward elevation with the elbow flexed to 90° stabilizing the scapula with one hand while applying a downward force on the distal forearm to create maximum internal rotation.

*Walch test* (posterior superior glenoid impingement): considered positive when pain is reproduced upon passively abducting the arm up to 150° while the arm is maintained in full external rotation [14–16].

#### Shoulder laxity

*Sulcus sign* applying inferior traction to the arm. Laxity is demonstrated by visible widening of the subacromial space with a sulcus appearing in the adjacent area just distal to the lateral acromion.

#### Nerves assessment

*Forward flexion test* (Axillary nerve): considered positive when there is a reduction of strength in forward flexion of the shoulder while the elbow is extended and the back of the hand is facing upward (the examiner standing in front of the patient and applying a downward force to the arm) [17].

*Roger-Bikelas-De Sèze manoeuvre*: (cervical radiculopathy) With the abducting shoulder of 90° the elbow extends while simultaneously pushes the head towards the opposite side: it is positive if it causes nervous brachial symptomatology [18, 19].

*Wall push-up test:* (long thoracic nerve) performed by asking the patient to do a push-up away from the wall while watching for winging of the scapula.

### Ultrasonographic evaluation

All the subjects underwent ultrasonographic examination (Samsung HM70A Ecograph), both static and dynamic, of both shoulders. All ultrasonographic exams were performed and interpreted by one experienced medical doctor operator (A.T.) (more than 10 years of experience in musculoskeletal sonography) who was blinded to the findings of the clinical examination. The ultrasonographic examination was performed with the patient seated with the operator sitting facing the patient using a linear small parts transducer (7.5–15 MHz).

The ultrasound examination provided for an evaluation of any injuries to the rotator cuff; Subacromial impingement and internal impingement was also assessed. The following diagnostic criteria were also used in interpreting the ultrasonographic images [20–24]:

*Impingement, anterior*: changes in the coraco-acromial ligament at rest (static imaging) or in forced abduction (dynamic imaging) with the ligament forming a convex superior arch.

*Internal impingement*: irregularity of the visible portion of the glenoid with the arm raised.

*Tendon degeneration*: coarse echogenicity of the tendon (hypoechoic with possible anechoic foci), hipomobility and diminished mobility and elasticity on dynamic imaging, enlarged.

*Calcific tendinopathy*: hyperechoic oval focus with or without sharp margins, posterior acoustic shadowing or posterior enhancement. With or without posterior acoustic shadowing or a sharp margin.

*Tendon inflammation*: anechoic area in the tendon, which represents acute inflammation when positive on Power Doppler and chronic inflammation when Power Doppler is negative. The diagnosis of the tendon inflammation is established as follows: inflammation describes an area that is compressible under the probe. During dynamic testing, the area generally diminishes in volume. By small movements of the probe in the sagittal plane, it is possible to see the whole tendon beneath the anechoic area. Surface irregularities of the outer portion appear hypoechoic and do not show a fibrillary appearance.

Elastography was also carried out to assess the degree of tissue elasticity. Elastography estimates the local longitudinal deformation of tissue through ultrasound. The degree of tissue elasticity is translated through a scale of colors, from blue to black, passing through yellow and red [25].

### Statistical analysis

All data were initially entered into an Excel database (Microsoft, Redmond, Washington—United States) and the analysis was performed using the Statistical Package for the Social Sciences Windows, version 21.0 (SPSS, Chicago, Illinois, USA). Descriptive statistics consisted of the mean ± standard deviation (SD) for parameters with Gaussian distributions (after confirmation with histograms and the Kolgomorov-Smirnov test), was performed with the ANOVA one-way for parametric variables while the Chi-square test or Fisher’s exact test (if cells < 5) for frequencies variables. A *p* value of < 0.05 was considered statistically significant.

## Results

No significant difference has been found between rugby players group and control group in age, height, weight body mass index (Table 1), demographic factors such as education, job-related physical demand, etc. (data not shown).

**Table 1:** Characteristics of the rugby players group and control group

	Rugby players	Control	*p*
*N*	26	26	
Age (years)	22.61 ± 1.6	22.15 ± 1.7	0.330*
Height (cm)	180.27 ± 6.4	177.53 ± 4.9	0.091*
Weight (kg)	80.35 ± 10.0	76.11 ± 8.3	0.104*
Body mass index (kg/m^2^)	24.71 ± 2.7	24.09 ± 1.86	0.345 *

Values are expressed as mean ± standard deviation

*ANOVA oneway

All rugby players had been playing for at least 6 years, for more than 6 h a week. All participants reported being right-side dominant except one rugby player and one control subject who were left-side dominant.

Table 2 shows physical examination results. None had a positive lift off test or Napoleon test. Only eight players were fully negative at the physical examination. In rugby players, the prevalence of Hawkins test, Sulcus sign and Roger-Bikelas-De Sèze manoeuvre were significantly higher compare to control (*p* < 0.05).

**Table 2:** Clinical evaluation

	Group	*N* (%)	*p*
Deltoid tenderness	Control	0 (0)	0.50
	Rugby players	1(3.8)	
Jobe test	Control	0 (0)	0.24
	Rugby players	2 7.7)	
Infraspinatus muscle strength test	Control	0 (0)	0.05
	Rugby players	4 (15.4)	
Speed test	Control	0 (0)	0.50
	Rugby players	1 (3.8)	
Forward flexion test	Control	0 (0)	0.50
	Rugby players	1 (3.8)	
Hawkins test	Control	0 (0)	0.01
	Rugby players	6 (23.1)	
Walch test	Control	0 (0)	0.24
	Rugby players	2 (7.7)	
Sulcus sign	Control	0 (0)	0.02
	Rugby players	5 (19.2)	
Wall push-up test	Control	0 (0)	0.5
	Rugby players	1 (3.8)	
Roger-Bikelas-De Sèze manoeuvre	Control	0 (0)	0.02
	Rugby players	5 (19.2)	
Shoulder pain needing pain-reliever drugs (0-six months)	Control	0 (0)	0.001
	Rugby players	10 (38.5)	

Ten (38.5%) rugby players reported at least a painful episode to the shoulder needing pain relief drugs in the previous 6 months (*p* = 0.001).

**Fig. 1:**
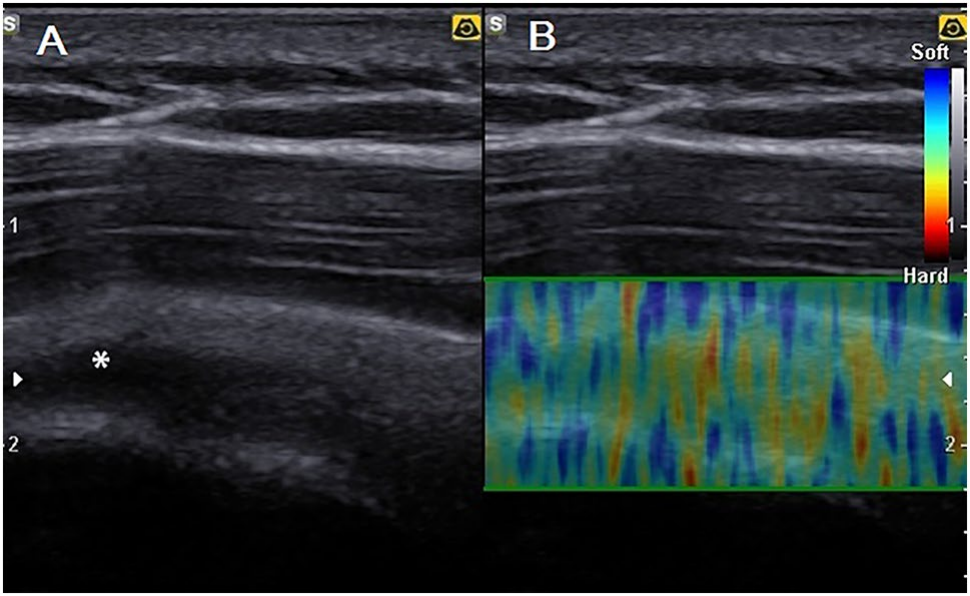
**A** An anechogenic area (*)—partial lesion—of the right supraspinatus tendon surrounded by an area of widespread hypoecogenicity—degeneration. **B** The elastography shows a poor elastic response of the tendon (widely green without responses to red and blue)

**Fig. 2:**
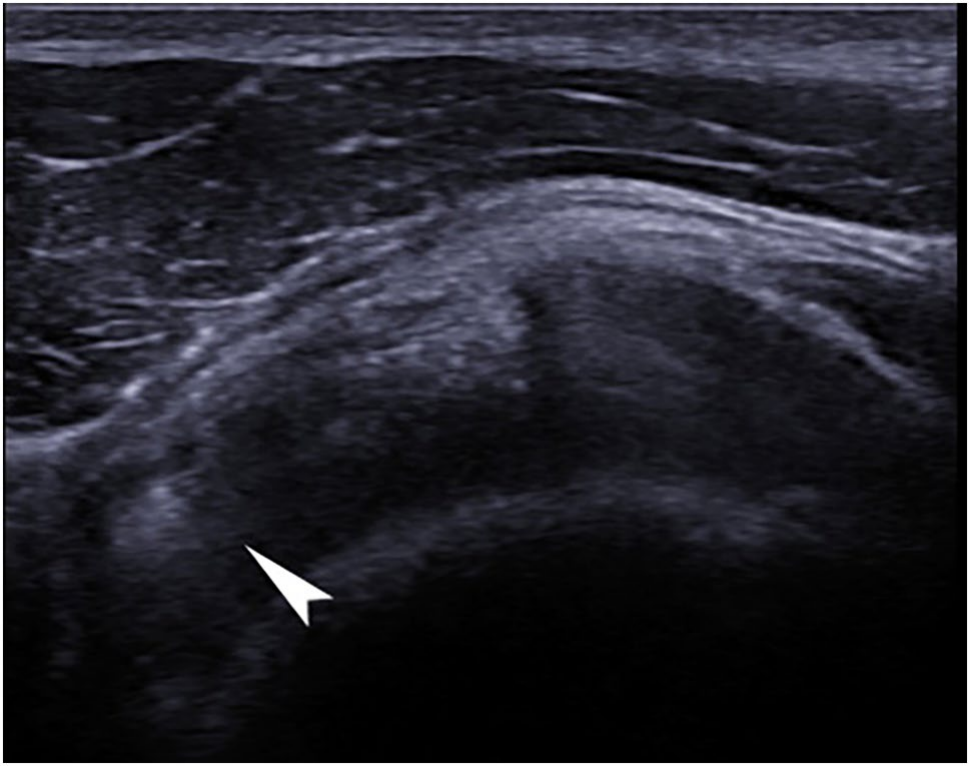
Arm in forced internal rotation, the probe on the coronal plane focusing on the right supraspinatus tendon. Ultrasonography shows hyperechoic formation with posterior enhancement (arrow)—calcification

Table 3 shows ultrasound finding. No changes of the long head of the biceps were present. The ultrasound examination of rugby players highlighted degenerative changes in supraspinatus tendons (Fig. 1) in six shoulders (*p* = 0.01), eight supraspinatus tendon calcification (Fig. 2) (*p* = 0.01), inflammation in tree shoulders (two supraspinatus tendons and one infraspinatous tendon), anterior impingement in two shoulders, internal impingement in one shoulder. In the control group, only one case of calcification and one case of anterior impingement were found. Eleven rugby players were fully negative.

**Table 3:** Ultrasonographic examination

	Group	*N* (%)	*p*
Supraspinatus tendon degeneration	Control	0 (0)	**0.01**
	Rugby players	6 (23.1)	
Supraspinatus tendon calcification	Control	1 (3.8)	**0.01**
	Rugby players	8 (30.8)	
Supraspinatus tendon inflammation	Control	0 (0.0)	0.18
	Rugby players	2 (7.7)	
Infraspinatus tendon inflammation	Control	0 (0.0)	0.50
	Rugby players	1 (3.8)	
Anterior impingement	Control	1(4.5)	0.50
	Rugby players	2 (7.7)	
Internal impingement	Control	0 (0.0)	0.50
	Rugby players	1 (3.8)

## Discussion

In the literature, there are few clinical-ultrasonographic studies on the athlete’ shoulder: to our knowledge, there is no retrospective study with a case control over rugby players.

In rugby players, both shoulders suffer continuous collision and stress during the game and training: although athletes seem to be asymptomatic, this sum of stresses involves joint changes in reported uninjured shoulder. Overall, in our study the shoulders of the rugby players show a greater number of ultrasound and clinical findings than the control group.

23.1% of rugby players show ultrasound findings of supraspinatus tendon degeneration and clinical tests suggestive of subacromial impingement (*p* = 0.01).

No ultrasound degeneration changes or clinical signs of rotator cuff tendon injuries have found in the control group.

The accuracy of ultrasonography for complete rotator cuff lesion is similar to magnetic resonance imaging while it has less sensitivity for partial lesion and impingement condition [26]. In young asymptomatic people full –thickness or partial-thickness tear of the rotator cuff are uncommon. No full -thickness lesion of the rotator cuff was found in the examined subjects; ultrasound changes such as anterior impingement do not show statistically significant differences between groups.

We hypothesize that some abnormal tendon findings on ultrasound images can be temporary changes, because of multiple shoulder impacts suffered during the game or training, while other findings can be a condition intended to evolve afterwards in more serious damage.

We found five positive sulcus sign in rugby players and no one in the control group. Although a positive sulcus sign may be an expression of a non-pathological ligament laxity, this condition could be associated with an early dysfunction of the shoulder static and dynamic stabilizers. Indeed, the rotator cuff represents an important dynamic stabilizer of the glenohumeral joint and electromyographic and cadaver studies have shown the external rotators contribute to anterior stability [27].

Partner et al. [28] in a study based on a questionnaire (Rugby Shoulder Score) filled out by a group of 86 uninjured rugby players, professionals and amateurs, noted that 55% of them reported some grade dysfunction of the shoulder.

Rugby players are constantly subject to painful syndromes, and their support staff continuously designs new methods to get pain relief [29]. In the rugby group, 10 players (38.5%) reported having had painful episodes to the shoulder needing pain relief drugs in the previous six months (*p* = 0.001).

Athletes who suffer a high number of shoulder collisions are familiar to pain. Rugby players can perceive shoulder pain or soreness as ordinary. They may underestimate symptoms, which instead must be carefully evaluated by the staff who take care of them, even to know when the athlete needs deepening instrumental exams. Besides, amateur players have less chances than professional players do, to be monitored by a dedicated medical staff; pathological conditions of their shoulder can persist without diagnosis and therapy.

Although the small number of participants, that suggests caution in data interpretation, this research display that uninjured shoulder of rugby players show joint abnormal findings which are not detected in the control group. These changes may come gradually before more severe shoulder lesions, which can impacts on the sport performance.

This investigation could allow identifying new elements to evaluate athlete's return to safe training and racing after shoulder pain occurrence.

As reported by Hemelryck et al. [30], 5% of rugby injuries affect the cervical spine: the nerve roots stress can be the basis of a relationships changes between scapula and caput humeri [31].

Roger-Bikelas-de Sèze manoeuvre is performed to distinguish a cervical neuropathic origin of shoulder pain: five rugby player reported paresthesia radiating up the examined limb. Based on our clinical experience, this finding is exceptional in young subjects without history of cervical spine pathology or recent cervical spine injury: this condition is worthy of careful investigation on a larger number of athletes.

The phenomenon of “stinger”, a neuropraxia of the cervical nerve root (s) or brachial plexus, is common among rugby players [7]. This can occur when the head is forced away from the shoulder and the shoulder is pushed downwards [32]. We assume that the a positive Roger-Bikelas-De Sèze maneuver reveal a silent pathological condition of the rugby player due to a cervical/brachial nerve roots dysfunction.

We believe that finding may provide a starting point to explore shoulder dysfunction of these athletes from a neurological point of view by making use of instrumental exams (that is electromyographic examination).

## Conclusions

Rugby players showed greater frequency of clinical signs suggestive of shoulder dysfunction and greater frequency of ultrasound changes suggesting supraspinatus tendon degeneration and calcification compared with the control group. The Roger-Bikelas-de Sèze maneuver may detect and monitor a subclinical irritation of cervical roots in those athletes.

Prospective studies with a higher number of participants could allow fixing the shoulder clinical and ultrasonographic changes to deepen or to keep watch with the goal to avoid worsened injuries.

## Data Availability

The datasets generated during and/or analysed during the current study are not publicly available, but are available from the corresponding author on reasonable request, upon approval by the local ethics committee.
